# Use of Baclofen for Alcohol Use Disorders in the United States

**DOI:** 10.3389/fpsyt.2018.00448

**Published:** 2018-09-21

**Authors:** James C. Garbutt

**Affiliations:** Department of Psychiatry, University of North Carolina at Chapel Hill, Chapel Hill, NC, United States

**Keywords:** Baclofen, alcohol use disorders, United States, clinical use, cirrhosis

The management of alcohol use disorders (AUDs) has been undergoing transformation since the discoveries of the effectiveness of naltrexone in the United States and acamprosate in Europe in the 1990's. These discoveries indicated that medications that target neurobiological processes can indeed have therapeutic value in AUDs. Prior to this there was only one effective medication for AUDs—disulfiram—with a mechanism of action targeted to the metabolism of acetaldehyde. The realization that medications that affect neurobiological processes can treat AUDs was truly a revolutionary finding and one that generated great hope that the treatment of AUDs would be based, at least in part, on a rational, scientific framework with, accordingly, improved effectiveness. Unfortunately, the intervening 20 years since these nodal discoveries has not seen a marked shift in the treatment of AUDs in the United States. Medications for AUDs are woefully under prescribed in the United States with estimates of only 5–10% of individuals with an AUD being offered an FDA approved medication on a backdrop of only 20% of AUD patients ever receiving any treatment (1). The reasons for this low rate of medication use are complex but appear to involve a lack of knowledge on the part of both clinicians and patients on the potential value of these medications coupled with concerns about tolerability and efficacy (2). Additionally, in the United States, the pharmaceutical industry is essentially not marketing any medication for AUDs that greatly reduces both clinician and patient awareness of medications for AUDs within the United States health care system.

The scientific community has approached the challenge of medication development for AUDs in two principal ways: (1) searching for new molecular targets connected with the underlying pathophysiology of AUDs with a goal of finding medications that may have efficacy for certain components of AUDs, e.g., protracted withdrawal, preoccupation, craving, with associated improvement in drinking behavior; (2) attempting to identify individual predictors of response to specific medications to enhance efficacy and move the field toward personalized medicine.

Baclofen represents a somewhat mature effort to target the GABA_B_ receptor—a novel target not addressed by other agents in the United States. The clinical evidence for efficacy and tolerability with baclofen for AUDs has grown considerably in the past 5 years but remains mixed with one meta-analysis showing evidence for efficacy for enhancing complete abstinence (3) and another (4) not finding clear evidence for an effect and a third showing evidence for reduction in drinking (5). This plays out in recent clinical trials where some show clear positive results and others no positive results using baclofen doses ranging in the 30–300 mg/d range. Furthermore, concerns with tolerability to baclofen, especially with sedation/drowsiness at higher doses (6), may deter clinicians and patients. Additionally, there is no effort on the part of the pharmaceutical industry to seek FDA approval for a baclofen formulation for AUDs in the United States. Thus, the use of baclofen in the U.S. is off-label. The upshot of all of this is that baclofen has a very low profile for the treatment of AUDs in the United States and there is minimal evidence of its use except by limited numbers of specialists. Accurate data on the use of baclofen for AUDs in the U.S. is lacking however, which makes it difficult to know with any precision how many clinicians have prescribed baclofen for AUDs.

One area that has attracted attention is the use of baclofen in patients with liver disease and an active AUD. The initial positive report of Addolorato et al. (7) that baclofen can increase rates of abstinence in patients with cirrhosis and improve liver profiles was noteworthy as it was the first evidence that a medication for AUD could be safe and effective in patients with liver disease. However, there has been limited clinical trial data to confirm this finding though a recent report by Morley et al. (8) is supportive. Because alcohol-induced liver disease is a significant cause of morbidity and mortality world-wide with nearly 500,000 yearly deaths (9), the further study of baclofen and other GABA_B_ receptor agonists for the treatment of AUDs in patients with liver disease is of obvious importance. However, to date, even within this population in the United States there appears to be limited use of baclofen.

From this writer's perspective, the use of baclofen for the treatment of AUDs in the United States faces two major impediments. First, the use of any medication to treat AUDs in the United States is very low. Second, the clinical trial data for baclofen is mixed which sends a complicated signal to clinicians. As additional baclofen trials are published it is hoped that evidence of efficacy becomes clearer and that some sense of who is the baclofen-responsive patient emerges along with guidance on dose and safety. Until this happens, it is unlikely that baclofen use for AUDs in the United States will expand. If new formulations of baclofen, such as R-baclofen or allosteric modulators of the GABA_B_ receptor, are shown to be effective for AUDs and are pursued by the pharmaceutical industry for FDA approval that would represent a significant positive step toward the use of GABA_B_ receptor agonists in the treatment of AUDs in the United States.

## Author contributions

The author confirms being the sole contributor of this work and has approved it for publication.

### Conflict of interest statement

The author declares that the research was conducted in the absence of any commercial or financial relationships that could be construed as a potential conflict of interest.
